# Self and caregiver report measurement of sensory features in autism spectrum disorder: a systematic review of psychometric properties

**DOI:** 10.1186/s11689-022-09473-7

**Published:** 2023-01-25

**Authors:** Jaclyn Gunderson, Emma Worthley, Breanne Byiers, Frank Symons, Jason Wolff

**Affiliations:** 1 Department of Educational Psychology, Educational Sciences Building College of Education and Human Development, University of Minnesota, 56 River Road, Minneapolis, MN 55455 USA; 2 Department of Psychiatry and Psychology, Mayo Clinic, 200 1St Street Southwest, Rochester, MN 55902 USA

**Keywords:** Autism spectrum disorder, Sensory, Assessment, Systematic review, Sensory responsivity

## Abstract

**Background:**

Unusual responses to sensory stimuli are considered a diagnostic symptom of autism spectrum disorder with mounting research efforts put towards understanding, characterizing, and treating such symptoms.

**Methods:**

This paper examines self and caregiver report tools used to measure sensory features in ASD through a systematic review of the psychometric evidence for their use. A total of 31 empirical papers were reviewed across 20 assessment tools. Substantial differences were identified in the specific sensory features defined across assessment tools. Sensory assessment questionnaires were evaluated against quality psychometric evidence criteria to provide a use recommendation.

**Results:**

Five assessments were identified to be “appropriate with conditions,” while no sensory assessment tools were identified to have sufficient quality psychometric evidence to provide a recommendation of “Appropriate” for measuring sensory features in ASD.

**Conclusion:**

Evidence from this review highlights potentially significant shortcomings among the current methods used to measure sensory features in ASD and suggests the need for more efforts in developing psychometrically sound sensory assessment tools for use in ASD populations.

**Supplementary Information:**

The online version contains supplementary material available at 10.1186/s11689-022-09473-7.

## Background

It is estimated that between 40 and 90% of individuals with ASD have significantly different behavioral responses to sensory experiences compared to typically developing peers [1–4]. Sensory differences are early emerging [5] and may have long-term effects on later functioning [6], making them ideal targets for early identification and intervention. The human sensory system is a complex biological system that, put simply is responsible for gathering information from the environment and relaying that information to the brain for responding [7]. While the focus of research is widely different across disciplines, researchers generally presume that neural differences in individuals with ASD leads to altered sensation, which results in atypical behavioral responses [8]. Henceforth, to broadly represent atypical responses to or interest in sensory stimuli, these behavioral responses will be referred to as *sensory features*.

Terms relating to sensory features in ASD vary and are poorly operationalized [9]. Hypo-reactivity, hyper-reactivity, and sensory seeking are widely used but synonymous terms, such as under/over-reactive, poor registration, high/low threshold, sensory sensitivity, under/over-reactivity, sensory craving, sensory interests, and sensory preoccupation are also found in the literature. Furthermore, terms such as sensory perception, sensory integration, multisensory integration, and sensory processing are common but refer to distinct constructs. Sensory perception refers to perceiving or becoming aware of sensory stimuli [10]. Sensory integration denotes how an individual organizes and uses sensory information [11], while multisensory integration refers to assimilation of spatially and temporally concurrent sensory stimuli [12]. Finally, sensory processing is described in the clinical literature (e.g., occupational therapy) as the overlap of becoming aware of stimuli and evoked behavioral responses [9, 13] while addressed in the neuroscience literature as mechanisms of sensation, transduction, and perception. The lack of common terms used to describe sensory features is problematic because differences in nomenclature influence the conceptualization and measurement of the construct.

Caregiver and self-report questionnaires are the most common tools used to measure sensory features in ASD, but there is limited understanding of their psychometric properties [14–16]. McConachie [16] evaluated the quality of psychometric evidence for three sensory assessment tools (Sensory Profile, Short Sensory Profile, and Sense and Self-Regulation Checklist) and found inadequate evidence of internal consistency, content, or structural validity, although they did document positive evidence of known group differences for all of the tools. DuBois and Lymer [15] found that caregiver and self-report questionnaires were used in 78.8% of the identified studies in a scoping review of sensory features in ASD. Of the 11 identified questionnaire measures, only seven had any published psychometric evidence. Burns [14] conducted a 20-year review of literature on sensory features in ASD and again found that nearly 70% of the studies utilized caregiver report questionnaires. Although the Burns review summarized the psychometric evidence for the identified tools, they did not appraise based on a quality criterion to anchor or make use recommendations. Taken together, prior evaluative work examining sensory measurement in ASD shows that caregiver and self-report tools are widely used while their psychometric properties are not well understood.

Studies performed with assessments with poor or unknown measurement properties are a waste of resources [17]. With the numerous caregiver and self-report sensory measurement tools currently available, determining their measurement properties is critical. This review aimed to critically appraise, compare, and summarize the quality of the measurement properties of sensory questionnaires for individuals with ASD by (a) identifying the specific sensory features/constructs measured, (b) examining the degree to which published studies provide evidence of different types of reliability and validity for each caregiver and self-report measure using standardized quality criteria, and (c) suggesting next steps related to the measurement of sensory features in ASD.

## Method

The following databases were searched for peer-reviewed papers published in English through June 2022*: PsychInfo, CINAHL, ERIC*, and *PubMed.* Each database was searched using the terms “autism spectrum disorder” or “autism” or “autistic disorder” and “sensory” and “measurement.” Sensory assessment tools were included in this review if they were found to be used in published literature with an ASD sample. Studies were included if they reported the development of an English caregiver or self-report sensory measurement tool or evaluated one or more measurement properties of an existing tool. Assessment tools examining single modality differences (i.e., vision, hearing, tactile) were excluded. Studies that tested research hypotheses about change or differences between groups but did not specifically evaluate the tool’s measurement properties were excluded. Measurement tools used in studies that included individuals who were being monitored for ASD symptoms even if they had another primary diagnosis (e.g., ASD symptoms in a fragile X population) were included.

**Fig. 1:**
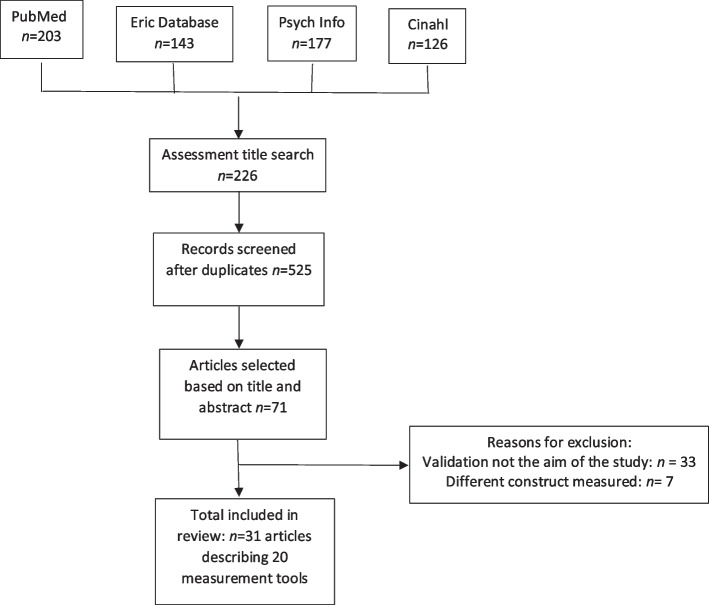
Included articles

The database search resulted in 649 articles that were imported into Rayyan-an online application for systematic reviews. Identified assessment tools were then individually searched for using the assessment tool name resulting in 226 articles. Duplicates were removed, resulting in 525 articles included in the title and abstract review. A total of 71 articles moved into the full-text review. An independent coder reviewed 20% of the articles with 98% overall agreement. The final study sample included 31 articles describing 20 measures. See Table S1 for included measures. Measures that had more than one published version update were evaluated together (e.g., SEQ 1.0, 2.1, 3.0), while measures that were modified for use with different demographic/age groups were evaluated individually (e.g., sensory profile, infant toddler sensory profile, adult and adolescent sensory profile). Three independent reviewers provided consensus agreement for the quality criterion score and use recommendation of each caregiver report measure. See Fig. 1 for the PRISMA flow diagram of included articles [18].

The COSMIN guidelines for the systematic review of patient-reported outcome measures were adapted for use in the current article [17]. Articles were coded for general design of the measure (what was being measured and in what population), content validity, structural validity, internal consistency, test–retest reliability, inter-rater reliability, and convergent validity. Quality criteria were adapted from the COSMIN manual and literature on validating scales used for health and social behavior [19]. Ratings of content validity considered if a method was reported to ask patients/caregivers and professionals about the relevance, comprehensiveness, and comprehensibility of the items in the questionnaire. Content validity also considered the number of individuals/caregivers and professionals that the items were tested on. If no methods were described, and/or participant or professional perspectives were not considered, the content validity was rated as unknown due to methodology. Further quality criterion details are listed in Table 1.

**Table 1:** Quality criterion for measurement properties adapted from COSMIN manual for systematic reviews of PROMS (Prinsen et al., [17])

Psychometric property	Rating	Quality criteria
Internal consistency	+	Cronbach’s alpha(s) ≥ 0.80
	?	Cronbach’s alpha not determined or dimensionality unknown
	-	Cronbach’s alpha (s) < 0.80
Reliability	+	ICC/weighted kappa ≥ 0.70 or Pearson’s *r* ≥ 0.80
	?	Neither ICC/weighted kappa, nor Pearson’s *r* determined
	-	ICC/weighted kappa < 0.70 or Pearson’s *r* < 0.80
Content validity	+	Process was described to consider how each item is relevant to the construct. All items are considered to be comprehensible, for the target population, and for the purpose of the measurement and the questionnaire is considered to be comprehensive
	?	Not enough information available; no research
	-	No process described for analysis or not all items are considered to be relevant, comprehensible or comprehensive for the construct measured
Structural validity	+	EFA: Factors should explain at least 50% of the variance CFA: RMSEA ≤ 0.06, CFI or TLI ≥ 0.95
	?	Explained variance not mentioned
	-	EFA: Factors explain < 50% of the variance; CFA: RMSEA > 0.06, CFI or TLI < 0.95
Convergent validity	+	Relationship has been examined between scale scores and similar constructs using multi-trait multi method matrix, latent variable modeling, or Pearson’s product moment coefficient, correlation coefficient ≥ 0.70
	?	Not enough information available
	-	Correlation with another scale measuring the same construct < 0.70

+  sufficient evidence; ? unknown, owing to poor methodological quality, not enough information, mixed results across studies;—contrary evidence. *AUC* Area under the curve, *CFA* Confirmatory factor analysis, *CFI* Comparative fit index, *EFA* Exploratory factor analysis, *ICC* Intraclass correlation coefficient, *LOA* Limits of agreement, *MIC* Minimal important change, *RMSEA* Root–mean–square error of approximation, *SDC* Smallest detectable change, *TLI* Tucker–Lewis fit index

Results of measurement properties evaluated within an article were rated against the quality criteria, and a grade was applied (+ / − /?). Results were pooled if two or more studies evaluated the same measurement property for a selected instrument. A rating of “ + ” indicated that the quality criterion was sufficiently met. Psychometric evidence that did not meet the quality criterion cut-off was marked with a “ − .” A rating of “ + / − ” indicated that quality criterion was met for some, but not all, subscales of the measurement tool. A rating of “?” was given if the grade was indeterminate owing to low methodological quality, not enough information (e.g., sample did not include autistic individuals), or mixed results across studies.

The psychometric evidence from included articles was synthesized to provide a general use recommendation. See Table 2 for recommendation criteria definitions adapted from Lecavalier et al. [20]. Assessment tools were classified as follows: (a) appropriate, (b) appropriate with conditions, (c) unsupported/insufficient, or (d) inappropriate. A recommendation of “appropriate” indicates quality criteria had been met for all relevant indices within the current review. A rating of “appropriate with conditions” indicates that sufficient psychometric quality criteria evidence existed in at least three indices. “Unsupported/insufficient” indicates that psychometric quality evidence is inconsistent, unavailable, or limited in the ASD population. The rating of “inappropriate” reflects psychometric criterion has been evaluated and is contrary to meeting the quality criterion in one or more indices of reliability or validity.

**Table 2:** Criteria for recommended use adapted from Lecavalier et al. [20]

Recommendation	Psychometric evidence
Appropriate	Sufficient psychometric evidence for reliability and validity in the ASD population with information available on **all** relevant indices
Appropriate with conditions	Sufficient psychometric evidence for reliability and validity for **some (at least 3)** but not all indices with an ASD sample population
Unsupported/insufficient	**Emerging data** showing sufficient evidence for reliability or validity in one or two indices in the ASD population or evidence in a group other than ASD (e.g., typically developing) but unknown in ASD population
Inappropriate	**Contrary data** on reliability or validity in one or more indices

## Results

A total of 31 articles across 20 sensory assessment tools were included in this systematic review. Studies were published from 1994 to 2022. Across the 20 assessment tools identified, 12 were caregiver report measures (Sensory Profile-SP, Short Sensory Profile-SSP, Sensory Sensitivity Questionnaire-Revised-SSQ-R, Infant Toddler Sensory Profile-ITSP, Sensory Behavior Schedule-SBS, Sensory Experiences Questionnaire-SEQ, Sensory Processing Measure-SPM, Sense and Self Regulation Checklist-SSC, Sensory Processing Self-Regulation Checklist-English-SPSRC, Sensory Assessment for Neurodevelopmental Disorders-SAND, Sensory Behavior Questionnaire-SBQ, Sensory Processing Scales Inventory-SP-Scales Inventory) [21–32]. Five were self-report measures (Adult and Adolescent Sensory Profile-AASP, Glasgow Sensory Questionnaire-GSQ, Sensory Processing Quotient-SPQ, Sensory Reactivity in Autism Spectrum-SR-AS, Sensory Sensitivities Scales-SeSS) [33–37]. Additionally, three measures provided a choice between caregiver report or self-report (Sensory Sensitivity Questionnaire-SSQ, Sensory Over Responsivity Inventory-SensOR, Brain Body Center Sensory Scale-BBCSS) [38–40]. Three assessments combined proxy report with an observational component (SAND, SensOR, and SP Scales Inventory as part of the SP3D) [24, 32, 39]. For this review, the observational components were not included in the psychometric evaluation.

### Constructs measured

Table S1 describes each assessment identified and the subscales and modalities targeted. Original terminology was retained to describe the scales and subscales of each assessment. For comparability, broad construct terms were utilized and based on the published descriptions and language used in the assessment. Four broad groups of sensory constructs were identified as sensory processing, sensory reactivity, unusual sensory behavior, and basic sensory detection. Seven assessment tools focused on measuring sensory processing (SP, SSP, AASP, ITSP, SPM, SPSRC-English, SP-Scales Inventory) [21, 22, 25, 27, 30, 32, 37]. Seven focused on sensory reactivity based on general awareness of and reaction to sensory stimuli (SSQ-R, SEQ, SSQ, GSQ, SR-AS, SAND, BBCSS) [24, 28, 31, 34, 36, 38, 40]. Four focused on unusual sensory behaviors in response to sensory stimuli (SBS, SSC the SBQ, SensOR) [23, 26, 29, 39]. Additionally two assessment tools were designed to measure an individual’s basic sensory detection and discrimination abilities (SPQ, SeSS) [33, 35].

### Psychometric quality

Table S2 displays the summary of the psychometric evidence quality for each of the sensory measures. No assessment tool was found to have evidence across all indices of reliability and validity used for this review. Therefore, no tool met the criteria to be recommended as “appropriate” for measuring sensory features in individuals with ASD. Five assessment tools were classified as “appropriate with conditions” (SEQ, SAND, SPQ, BBCSS, SR-AS) [24, 28, 34, 35, 40–43]. Six assessments were identified as “inappropriate,” indicating evidence contrary to the quality criteria in one or more indices of reliability or validity (SSP, SSQ-R, AASP, SBS, SSQ, SSC) [26, 29, 31, 37, 38, 44, 45]. Nine assessment tools were rated as “unsupported/insufficient,” indicating limited or inconsistent data on reliability and validity with the ASD population (SP, ITSP, SPM, SensOR, GSQ, SPSRC-English, SBQ, SeSS, SP-Scales Inventory) [21, 23, 30, 32, 33, 36, 39, 46–57].

## Discussion

Appropriate measurement is critical for advancing our understanding of sensory features in ASD. The purpose of this review was to evaluate how sensory features in ASD are currently being measured in caregiver and self report questionnaires and evaluate the psychometric evidence of the tools. Of the 20 sensory measures identified, none met all quality criteria for use in measuring sensory features in ASD, and only five were rated as “appropriate with conditions.” The Sensory Experiences Questionnaire is recommended for measuring sensory reactivity features in children with ASD between the ages of 2–12 years. This recommendation is supported by quality criterion evidence for internal consistency, test–retest reliability, and a CFA demonstrating satisfactory structural validity [28, 42, 43]. The psychometric properties were assessed with sufficient sample sizes including individuals with ASD. The SR-AS is recommended for measuring sensory reactivity features of ASD in autistic adults without cognitive impairments based on quality criterion in internal consistency, content validity, and structural validity [34]. The SAND caregiver interview, although deemed “appropriate with conditions” for measuring sensory reactivity in children warrants more research to validate the factor structure and its relationship with the SAND observational counterpart [24]. The BBCSS is deemed “appropriate with conditions” as a tool for measuring sensory reactivity features from childhood into adulthood (5–58 years old), yet further research with an ASD-specific sample would strengthen this recommendation [40]. The Sensory Processing Quotient is recommended as “appropriate with conditions” for measuring basic sensory detection in autistic adults [35].

Evidence from this review highlights significant shortcomings of current methods used to measure sensory features in ASD. While Burns et al. [14] pointed out the lack of psychometric evidence of sensory assessment tools generally, the present review adds to our understanding by identifying the specific gaps in psychometric evidence for these tools [19]. Content validity is arguably the most critical measurement property: items that are not relevant, comprehensive, and clear do not contribute meaningful information. The current results indicate that most assessments (18 out of 20) used to measure sensory features in ASD do not meet quality criterion evidence for content validity based on COSMIN recommendations [17, 19]. Only two assessments included in the review (SR-AS, SPQ) described the process used to ask autistic adults or caregivers and professionals about the relevance, comprehensiveness and comprehensibility of items [34, 35]. The SensOR and ITSP [30, 39] described a process for evaluating content validity but did not test the items with an ASD population. In other studies content validity was assumed (e.g., Talay-Ongan et al., [31]) or only based on literature reviews without an evaluation of the items used in the assessment (e.g., Harrison & Hare, [29]; Minshew et al., [38]; Robertson & Simmons, [36]).

The results of the current review show substantial variability across dimensions of sensory being sampled for in the items of sensory questionnaires. Overall, the primary goal of behavioral assessments is to obtain data from functional items that meaningfully underscore a single or multidimensional domain and contribute significantly to the construct. Structural validity refers to the degree to which an assessment reflects the dimensionality of focal constructs and how the items are interrelated [17]. Only four of the included measures had positive published evidence of structural validity (SEQ, SPQ, BBCSS, SR-AS) [34, 35, 40, 43]. For this discussion, construct terms were made consistent for comparability while original terminology was retained in Table S1. For example, the authors of the SEQ, confirmed its primary factor structure, with four sensory response patterns (hypo-reactivity, hyper-reactivity, sensory seeking, enhanced perception) and alternative structures broken down by modality [43]. In contrast, items from the BBCSS were found to load onto eight unidimensional subscales, including “auditory threat hypersensitivity,” “auditory hyposensitivity to voices,” “visual hypersensitivity,” “tactile hypersensitivity,” “affiliative touch aversion,” “selective eating,” “ingestion problems,” and “digestive problems” [40]. The SR-AS assessment loaded onto four factors including high awareness/hyper-reactivity, low awareness/hypo-reactivity, sensory interest, and sensory/ motor [34]. Yet the SPQ was evaluated with a unidimensional structure [35]. The SEQ and SR-AS sensory dimensions correspond with DSM-5 symptomology of ASD to include hyper- or hyporeactivity to sensory input and unusual interests in sensory aspects of the environment. However, as Kolacz et al. [40] point out, the presence of both hypersensitivity and hyposensitivity in ASD may suggest that atypical reactivity to sensory stimulation may be contextual rather than uniformly characterized by a higher/lower sensitivity of the sensory system receptors and/or perceptual systems. Additionally, Thye et al. [58] linked specific sensory modality responses (visual, auditory, tactile, olfactory/gustatory) vs. response patterns (hypo or hyper) with social deficit symptoms of ASD. Taken together, these results raise the question of whether the validity of sensory features research in ASD rests on arbitrary decisions regarding labels used to organize concepts [48]. In other words, we may not yet have determined how to definitively catalog and discriminate the relevant sensory features.

Furthermore, evidence of structural validity is a prerequisite for interpretations of internal consistency [19]. However, five measures included in the review were evaluated for internal consistency without consideration for structural validity (SSQ-R, SSC, SPSRC, SAND, SBQ) [23–26, 31]. Existing and future sensory measures in ASD must prioritize structural validity. Many sensory questionnaires have been criticized for being too limited across modalities and broad in scope [2, 39, 45, 59]. For example, the SPM [27] broadly contains scales such as Social Participation, Body Awareness, Balance and Motion, and Planning and Ideas. Conversely, the SBS [29] includes 17 items across five modalities leaving single items representing full domains such as auditory responding captured by “person makes unusual vocalizations.” Likewise, the SSQ [38] used 13 items to capture information across four domains of sensory features. Consequently, without clear, consistent constructs and agreement on their meaning and measurement, the field is limited in our scientific understanding and ability to improve therapeutic outcomes related to sensory features in ASD.

**Fig. 2:**
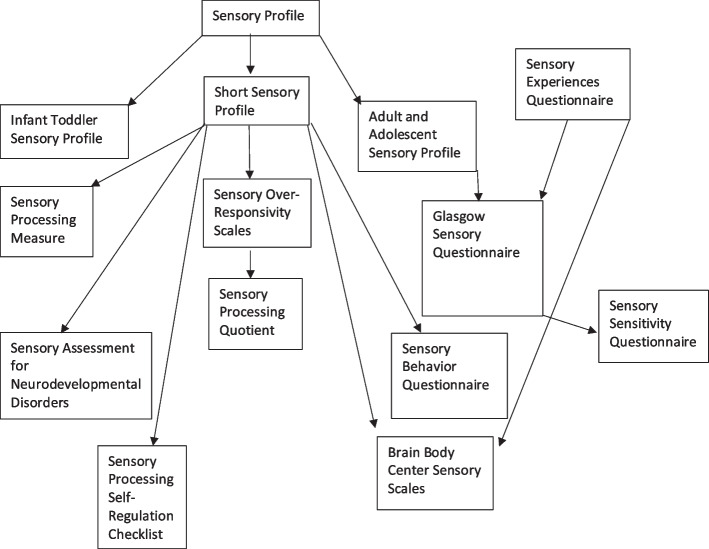
Pathways of convergent validity estimates

While no “gold standard” sensory measure exists, many researchers examined convergent validity via correlations with scores collected from a source assumed to measure the same “construct,” most commonly the SP or SSP. According to Prinsen et al. [17] when comparing convergent validity, one must consider the clarity of the construct measured by the comparator instrument and determine if the comparator instrument itself has “sufficient” psychometric properties. This review identified eight measures that were assessed in comparison to the different versions of the SP including the SSP, ITSP, and AASP (See Fig. 2). Overall, drawing inferences about convergent validity evidence for these assessment tools is strongly cautioned due to the weak psychometric evidence of the comparator tool for use in an ASD population [60].

### Future directions

The call for advancements in measuring sensory features is not new [1, 14, 15, 61, 62]. However, evidence from the current review suggests the need to create more orthogonal assessment strategies with clearer operationalizations of the specific constructs being measured. The research domain criteria (RDoC) proposed by the National Institutes of Mental Health (NIMH) is a research framework that may be useful in advancing this agenda for sensory features in ASD [63]. RDoC is a framework cutting across six major domains of human functioning along the continuum of normal to abnormal based on the intersection of information from genetics, biology, and behavior. Researchers should consider more fine-grained measurement of sensory features through the lens of the RDoC framework in which behavioral elements, processes, mechanisms, and responses are considered. Uljarević et al. [64] argued that classifying the fundamental aspects of sensory features in ASD and identifying their genetic, neural, and behavioral correlations across individuals is a necessary prerequisite to identifying meaningful treatment targets. To achieve this, they urged for research into sources of variability in sensory features between individuals through a three-pronged approach that (a) considers sensory features as dimensional constructs, (b) examines individual differences, and (c) moves to comprehensive, multidimensional, and multimodal approaches to the measurement of sensory features. However, any such measures require rigorous development and validation.

Although research consistently demonstrates that individuals with ASD score differently than typically developing individuals on evaluations of sensory features [1, 4, 65–68], it is time to hone in on measuring sensory features more comprehensively and clearly. One solution may be to bring together experts across disciplines (psychology, occupational therapy, neuroscience) to come to consensus around the dimensions of sensory features as it pertains to ASD. Overall, we must classify the fundamental bounds of sensory features as a construct related to ASD and test theoretical models to provide a better outline for the construct dimensions. The field continues to see therapies that claim to treat sensory symptoms central to ASD without discretely measuring the symptom or monitoring change in the behavior [41, 42, 61]. Valid and reliable sensory measures are critical to ensuring therapeutic outcomes map onto the claims of the intervention.

Finally, we believe that to advance measurement of sensory features as a behavioral outcome in treatment trials, assessments need to be evaluated for sensitivity to change. Responsiveness refers to a measure's ability to detect change over time in the construct in question, moreover validity in a change of score (e.g., after treatment) [17]. Testing an instrument’s responsiveness can be completed with comparisons to a “gold standard” score over time via correlations, an assessment of sensitivity and specificity, or hypothesis testing in conjunction with a comparison instrument. However, none of the assessments examined in this review reported this measurement property. It is important to the future of intervention work that measurement instruments be responsive to meaningful changes in the sensory features.

### Limitations

While caregiver and self report questionnaires are the most commonly used assessments in studies of sensory features in ASD [14], other types of assessments should also be evaluated, such as observational and performance-based measures. For example, the SensOR and its modified questionnaire component the SP-Scales Inventory have been updated into the SP3D which includes a performance based assessment counterpart [32, 39, 69]. This is also the case for the SAND [24]. Psychometric properties of such direct assessments must be specifically evaluated and not assumed. It is important to note that this review was limited to peer-reviewed evidence of internal consistency, test-retest reliability, content validity, structural validity, and convergent validity. However, other measurement properties, such as those related to hypothesis testing, could provide additional evidence towards construct validity. Known group differences of sensory features in individuals with ASD compared to the general population is one such area that has been repeatedly reported. Additionally, while recommendation scores in the current review treat each psychometric criteria domain equally, we emphasize the importance of content validity and structural validity in interpreting the psychometric evidence from other reliabilability and validity evaluations.

## Conclusion

Sensory features are a prominent symptom identified in individuals with ASD. Caregiver and self-report questionnaires have long served as the primay mode for measuring sensory features. Employing the COSMIN guidelines for rigorous evaluation of psychometric quality, we failed to identify any current measure that met sufficient quality criterion across all included domains of psychometric evidence. Overall, our results suggest that measurement of sensory features in ASD relies largely on questionnaires not validated with an ASD sample. However, the SEQ, SAND, SPQ, BBCSS, and the SR-AS showed sufficient quality criterion across at least three domains of psychometric evidence. The SEQ holds promise as a caregiver report sensory measure for children with ASD. The SR-AS is recommended as a measure with potential utility for measuring sensory features via self-report in adults and the SPQ for measuring basic sensory detection. Yet the lack of consensus around terminology and components relevant to sensory functioning are barriers to advancing the field. For this reason, we recommend a return to the basics in best practices for developing and validating scales.

## Supplementary Information

**Additional file 1: Table S1.** Summary of Measures Used to Assess Sensory Features in Individuals with ASD. Table (larger than 1 page) including assessment tool names, description, target population, administration type.

**Additional file 2: Table S2.** Summary of Psychometric Quality of Self and Caregiver-Report Sensory Measures Used in ASD. Table (landscape layout) including caregiver report assessment tool names, and quality criterion score for psychometric properties including internal consistency, test-rerest reliability, inter-rater reliability, content validity, structural validity, and convergent validity, and use recommendation.

## Data Availability

Data generated for this article are available from the corresponding author on reasonable request.

## Abbreviations

ASD: Autism spectrum disorder
RDoC: Research domain criteria
COSMIN: COnsensus-based Standards for the selection of health Measurement Instruments
PRISMA: Preferred Reporting Items for Systematic Reviews and Meta-Analyses
AUC: Area under the curve
CFA: Confirmatory factor analysis
CFI: Comparative fit index
EFA: Exploratory factor analysis
ICC: Intraclass correlation coefficient
LOA: Limits of agreement
RMSEA: Root–mean–square error of approximation
SDC: Smallest detectable change
TLI: Tucker–Lewis fit index
