# Supplementary material for: Self and caregiver report measurement of sensory features in autism spectrum disorder: a systematic review of psychometric properties
Source: J Neurodev Disord. 2023 Jan 25;15:5. doi: 10.1186/s11689-022-09473-7 (PMC9875408; doi:10.1186/s11689-022-09473-7)
Supplement: Supplementary file 1 — Additional file 1: Table S1. Summary of Measures Used to Assess Sensory Features in Individuals with ASD. Table (larger than 1 page) including assessment tool names, description, target population, administration type. [file 11689_2022_9473_MOESM1_ESM.docx]

**Table 1**

*Summary of Measures Used to Assess Sensory Features in Individuals with ASD*

| **Assessment Tool** | **Description** | **Constructs/Subscales** | **Target**  **Population** | **Item/**  **Response** | **Admin** |
| --- | --- | --- | --- | --- | --- |
| Sensory Profile (SP; Dunn, 1994) | Measures sensory features based on Dunn’s Model of Sensory Processing. The four-quadrant model considers sensory threshold and the continuum of behavior responses. The questionnaire is grouped into three main sections: Sensory Processing, Modulation and Behavioral/  Emotional Responses. | Sensory processing. Sensory seeking, sensation avoiding, sensory sensitivity, and low registration. Auditory, visual, taste/smell, movement, body position, touch, and emotional/social. | 3-10 years | 99 items, updated version 125 items; 5-point Likert scale | Caregiver |
| Short Sensory Profile (SSP; McIntosh et al., 1999) | Based on Dunn’s Model of Sensory Processing and designed to assess  behavioral responses to everyday sensory stimuli. Modified from the Sensory Profile to create an abbreviated version. | Sensory processing. Tactile sensitivity, taste/smell sensitivity, movement sensitivity, under-responsive/seeks sensation, auditory filtering, low energy/ weak, and visual auditory sensitivity. | 3-14 years | 38 items; 5-point Likert scale | Caregiver |
| Sensory Sensitivity Questionnaire-Revised (SSQ-R; Talay-Ongan et al., 2000) | Measures sensory sensitivity to stimuli based on perception and response style. | Sensory reactivity. Hypo and hyper responses to auditory, tactile, visual, gustatory, vestibular, and olfactory domains | Children | 45 items; Yes/No | Caregiver |
| Adult and Adolescent Sensory profile (AASP; Brown et al., 2001) | An extension of Dunn’s Model of Sensory Processing for adults. Intended to capture information about behavioral responses to everyday sensory stimuli. | Sensory processing. Sensory seeking, emotional reactive, low endurance tone, oral sensitive/sensitivity, inattention distractibility, poor registration, sensory sensitivity, sedentary, and fine motor perceptual. | 11+ years | 60 items; 5-point Likert scale | Self-Report |

| Infant Toddler Sensory Profile (ITSP: Dunn & Daniels, 2002) | Measures responses to sensory events in daily life based on Dunn’s Model of Sensory Processing. Considers sensory processing, modulation, and behavioral responses. | Sensory processing. General processing, auditory, visual, touch, movement, and oral sensory processing categorized as sensation seeking, low registration, sensory sensation or sensation avoiding. | 7-36 months | 48 items; 5-point Likert scale | Caregiver |
| --- | --- | --- | --- | --- | --- |
| Sensory Behavior Schedule (SBS; Harrison & Hare, 2004) | Measures inappropriate and/or  markedly dysfunctional overt behaviors associated with sensory difference specific to ASD. | Unusual sensory behavior. Visual, auditory, gustatory, olfactory, tactile, vestibular movement, proprioception, temperature. | Adults | 17 items | Caregiver |
| Sensory Experiences Questionnaire (SEQ, versions 1.0. 2.0. 2.1, 3.0; Baranek et al., 2006) | Characterizes sensory features of autism, discriminating patterns of hypo- and hyper-responsiveness based on Optimal Band Theory which considers sensory responses stemming from orienting threshold and sensory aversion threshold. Intended to capture the frequency of sensory response behaviors across social and nonsocial contexts. | Sensory reactivity. Hypo/ hyperresponsiveness, sensory interests, repetitions and seeking behaviors, and enhanced perception across social and nonsocial domains, and auditory, visual, tactile, gustatory/olfactory, vestibular, proprioceptive modalities. | SEQ 1.0 and 2.1=6 months – 6 years; SEQ 3.0 =  2–12 years | SEQ= 43; SEQ 2.1=33 items; SEQ 3.0=105 items; 5-point Likert scale | Caregiver |
| Sensory Processing Measure (SPM; Parham et al., 2007) | Examines behaviors associated with sensory processing across environments such as home, classroom, and school based on Ayres’ sensory integration theory. | Sensory processing. Social participation, vision, hearing, touch, body awareness, balance/motion, planning and ideas, and total sensory systems. | 5-12 years old | 75 items; 4-point Likert scale | Caregiver |
| Sensory Sensitivity Questionnaire (SSQ; Minshew & Hobson, 2008) | Measures subjective sensory experiences and common reactions to sensory stimuli reported by individuals with ASD. | Sensory reactivity. Auditory, light, tactile and temperature sensitivities, pain tolerance, awareness of smell or taste; Low temperature/ pain tolerance, high temperature/ pain tolerance, tactile, overall sensory sensitivities. | High functioning autistic individuals > 8 | 13 items; yes/no | Caregiver or self-report |

| Sensory Over Responsivity Scales (SensOR); Schoen et al., 2008) | Measures sensory over-responsivity based on the Arousal Theory. Combines an observational performance measure and questionnaire in either caregiver-report or self-report (for adults) format. | Unusual sensory behavior. Sensory over-responsivity across visual-olfactory, auditory, proprioception-movement, tactile, food. | 3 years-Adults | 76 items; Yes/No score + direct observ-ation | Caregiver or self- report |
| --- | --- | --- | --- | --- | --- |
| Glasgow Sensory Questionnaire (GSQ; Robertson & Simmons, 2012) | Measures sensory symptoms commonly associated with ASD in the general population based on sensory signs and symptoms reported in the literature and by parents of children with autism. | Sensory reactivity. Hyper- and hypo-sensitivities; visual, auditory, gustatory, olfactory, tactile, vestibular, and proprioceptive stimuli. | Adolescent and Adults | 42 items; 5-point Likert scale | Self-report |
| Sense and Self-Regulation Checklist (SSC; Silva & Schalock, 2012) | Measures self-regulation and abnormal sensory responses to ordinary injurious and non-injurious stimuli. Based on Five Phase theory of Chinese Medicine. | Unusual sensory behavior. Touch–pain, auditory, visual, taste–smell, hyperreactive to non-injurious stimuli, and hyporeactive to injurious stimuli. | Children < 6 | 4-point Likert scale | Caregiver |
| Sensory Processing Quotient (SPQ; Tavassoli et al., 2014) | Investigates basic sensory sensitivity, with no reference to affective response. Focuses on basic detection and/or discrimination abilities. | Basic sensory detection. Hyper-and hypo-sensitivities to tactile, auditory, olfactory, visual, and gustatory stimuli. | Adults | 35 items; 4 point scale | Self-report |
| Sensory Processing Self-Regulation Checklist-English (SPSRC; Lai & Chiu, 2019) | Examines self-regulation and sensory processing via parent report of children’s difficulties with behavioral regulation and behavioral responses to sensory input. | Sensory processing. Self-regulation; physiological, social, cognitive, emotional, and facing changes and challenges. Sensory seeking, under/over-responsivity, and stability of sensory responsivity; auditory, visual, tactile, gustatory/ olfactory, vestibular, and proprioceptive. | 3-8 years | 130 items; 5 point Likert scale | Caregiver |

| Sensory Reactivity in Autism Spectrum (SR-AS; Elwin et al., 2016) | Examines awareness and reactions to sensory stimuli including sensory reaction to stimuli inside the body. | Sensory reactivity. hyperreactivity/high awareness, sensory interest, sensory motor, hyporeactivity/low awareness  visual, auditory, gustatory, olfaction, tactile, vestibular, proprioception and interception. | High-functioning autistic adults | 32 items; 4-point Likert scale | Self-report |
| --- | --- | --- | --- | --- | --- |
| Sensory Assessment for Neurodevelopmental Disorders (SAND; Siper et al., 2017) | Characterizes sensory reactivity symptoms based on DSM-5 criteria for ASD. Combines a clinician-administered observation and caregiver interview. | Sensory reactivity. Hyper-, hypo-reactivity, and seeking in visual, auditory, and tactile modalities. | 2-12 years | 36 items; Yes/No present and severity + clinician observ-ation | Caregiver  interview |
| Sensory Behavior Questionnaire (SBQ; Neil et al., 2017) | A parent-report measure of the frequency and impact of sensory behaviors in autistic children and children with moderate-to-severe learning disability. | Unusual sensory behavior. Auditory, visual, vestibular/proprioceptive, tactile, and oral motor processing, general reactions, and organization. | 6-17 years | 50 items; 6-point Likert scale | Caregiver |
| Sensory Processing Scales Inventory (SP Scales Inventory; Schoen et al., 2017) | Caregiver report questionnaire built from the SensOR inventory (Schoen et al, 2008) to include under- responsive and sensory craving behavior. Now a component of the Sensory Processing 3 Dimensions Scale (SP3D; Mulligan et al., 2019).  Characterizes patterns of sensory modulation based on responsivity to sensory experiences in children with a developmental or intellectual disability. Designed to be paired with examiner-administered SP Scale Assessment (Schoen et al., 2014). | Sensory processing. Sensory over responsivity (touch/texture, sound, smell, vision, food-related, movement/vestibular/ proprioception), sensory under responsivity (touch, pain, sound, vision), sensory craving (touch/ sound, vision, oral, movement/ vestibular/ proprioception). | 4-18 years | 96 items; Yes/No | Caregiver |
| Brain Body Center Sensory Scale (BBCSS; Kolacz et al., 2018) | Examines patterns of responding to everyday sensory stimuli. Based on the Polyvagal Theory (evolutionary neurophysiological framework) to understand sensitivities to the sensory environment and reduce defense reactions. | Sensory reactivity. Auditory, visual, tactile, and ingestive/digestive; auditory threat hyperresponsivity, auditory hyposensitivity to voices, visual sensitivity, tactile hyperresponsivity, affiliative touch aversion, selective eating, ingestive problems, and digestive problems | 5-58 years | 55 items; 4-point Likert scale | Caregiver/self-report |
| Sensory Sensitivity Scales (SeSS; Aykan et al., 2020) | Examines sensory sensitivities  as separate domains and independent of social/emotional features using self-report. Based on sensory sensitivity considered as an aspect of sensory modulation within the Ayres Sensory Integration model. | Basic sensory detection. Auditory, visual, and somatosensory (touch/pressure, pain, itch) sensitivities | Adults | 35 items; 5-point Likert scale | Self-report |
