# Supplementary material for: Self and caregiver report measurement of sensory features in autism spectrum disorder: a systematic review of psychometric properties
Source: J Neurodev Disord. 2023 Jan 25;15:5. doi: 10.1186/s11689-022-09473-7 (PMC9875408; doi:10.1186/s11689-022-09473-7)
Supplement: Supplementary file 2 — Additional file 2: Table S2. Summary of Psychometric Quality of Self and Caregiver-Report Sensory Measures Used in ASD. Table (landscape layout) including caregiver report assessment tool names, and quality criterion score for psychometric properties including internal consistency, test-rerest reliability, inter-rater reliability, content validity, structural validity, and convergent validity, and use recommendation. [file 11689_2022_9473_MOESM2_ESM.docx]

**Table 4**

*Summary of Psychometric Quality of Self and Caregiver-Report Sensory Measures Used in ASD*

| **Assessment Tool**  **Study** | **Reference sample** | | | **Reliability** | | | **Validity** | | | **Recommendation** |
| --- | --- | --- | --- | --- | --- | --- | --- | --- | --- | --- |
|  | **Age range (years)** | **Sex**  **% Male** | **Sample (N) & diagnosis** | **Internal consistency** | **Test-retest** | **Inter-rater** | **Content** | **Structural** | **Convergent** |  |
| Sensory Profile (SP) |  |  |  |  |  |  |  |  |  | *Unsupported/*  *Insufficient*-No ASD sample, content validity assumed. |
| Dunn, 1994 | 3-10 | 68 | 64=TD |  |  |  | ? |  |  |  |
| Dunn & Brown, 1997 | 3-10 | 50 | 1115=TD | ? |  |  |  | ? |  |  |
| Ohl et al., 2012 | 3-6 | NR | 55=TD | ? | ? |  |  |  |  |  |
| Short Sensory Profile (SSP) |  |  |  |  |  |  |  |  |  | *Inappropriate*-Factor structure does not fit for ASD. |
| Williams et al., 2018 | 2-12 | 80 | 388=ASD |  |  |  |  | - |  |  |
| Sensory Sensitivity Questionnaire-Revised (SSQ-R) |  |  |  |  |  |  |  |  |  | *Inappropriate*-, content validity assumed; internal reliability tested with two repeated items. |
| Talay-Ongan et al., 2000 | 4-14 | 90 | 30=ASD; 30=TD |  | - |  | ? |  | ? |  |
| Adult and Adolescent Sensory Profile (AASP) |  |  |  |  |  |  |  |  |  | *Inappropriate*-No ASD sample, some subscale coefficients <.70, comprehensibility not evaluated, weak fit with original factor structure. No correlation of scores and thresholds. |
| Brown et al., 2001 | 17-79; Revised version=18-68 | 38; Revised version=43 | 615=TD; Revised version=  93 | +/- |  |  | ? | - |  |  |
| Schulz et al., 2021 | 17-29 | 33 | 118=TD |  |  |  |  |  | **-** |  |
| Infant Toddler Sensory Profile (ITSP) |  |  |  |  |  |  |  |  |  | *Unsupported/*  *Insufficient*-No ASD sample, limited sample size for convergent validity. |
| Dunn & Daniels, 2002 | Birth-3 | 51 | 401=TD | ? |  |  | ? | ? |  |  |
| Woodard et al., 2012 | 2-3 | 88 | 8=ASD; 8= TD |  |  |  |  |  | ? |  |
| Sensory Behavior Schedule (SBS) |  |  |  |  |  |  |  |  |  | *Inappropriate-* Reliability coefficient <.70, content validity based on literature review and no evaluations. |
| Harrison & Hare, 2004 | 20-50 | 76 | 25=ASD |  | - | - | ? |  |  |  |
| Sensory Experiences Questionnaire (SEQ) |  |  |  |  |  |  |  |  |  | *Appropriate with Conditions*- Reliability coefficients <.70 for some subscales, small test-retest sample, content validity based on literature review and no evaluations. Additional factor structure on TD sample. |
| Baranek et al., 2006 | 5 mos -6 years | 67 | 56=ASD; 24=PDD; 68=DD; 110=TD | +/- |  |  | ? |  |  |  |
| Little et al., 2011 | 6 mos-6 years | 68 | 109=AS; 83=DD; 163=TD | +/- | +/- |  |  |  |  |  |
| Ausderau et al., 2014 | 2-12 | 82 | 1307=  ASD |  |  |  |  | + |  |  |
| Lee et al., 2022 | 3 | 50 | 2195=TD |  |  |  |  | ? |  |  |
| Sensory Processing Measure (SPM) |  |  |  |  |  |  |  |  |  | *Unsupported/*  *Insufficient* – Correlation with SP-2, coefficient ranged between .32 and .79, with SP >.70. |
| Brown et al., 2010a/b | 5-10 | 47 | 30=TD mothers & fathers  19= TD teachers | ? | ? | ? |  |  | ? |  |
| Dugas et al., 2018 | 5-8 | 82 | 34=ASD |  |  |  |  |  | +/- |  |
| Brown et al., 2021 | 6-10 | 50 | 40=TD |  |  |  |  |  | ? |  |
| Sensory Sensitivity Questionnaire (SSQ) |  |  |  |  |  |  |  |  |  | *Inappropriate*-No evaluation for content validity based on literature, reliability coefficient <.70, no relations between reports of sensory abnormality  on the SSQ and sensory perceptual processing testing. |
| Minshew et al., 2008 | 8-54 | 83 | 60=ASD; 61=TD |  |  | - | ? |  | - |  |
| Sensory Over-Responsivity Inventory (SensOR) |  |  |  |  |  |  |  |  |  | *Unsupported/*  *Insufficient* – NO ASD sample, reliability coefficient <.70 for some subscales. |
| Schoen et al., 2008 | Sample 1=3-55; Sample 2=4-55 | NR | Sample 1,60=TD; 65=OR Sample 2, 44=TD; 48=OR | ? |  | ? |  | ? | ? |  |
| Glasgow Sensory Questionnaire (GSQ) |  |  |  |  |  |  |  |  |  | *Unsupported/ Insufficient* – undetermined ASD sample, content validity based on literature, correlation with AASP <.70 for participants with ASD |
| Robertson et al., 2013 | 16-66 | 33 | 212=TD | ? |  |  | ? |  |  |  |
| Horder et al., 2014 | NR | 28 | 749=TD;  23=ASD |  |  |  |  |  | - |  |
| Sense & Self-Regulation Checklist (SSC) |  |  |  |  |  |  |  |  |  | *Inappropriate* – test-retest reliability coefficient <.70, no method to ask experts about content |
| Silva & Schalock, 2012 | 2-6 | 63 | 99=ASD; 28=DD; 138=TD | +/- | - | ? |  |  |  |  |
| Sensory Processing Quotient (SPQ) |  |  |  |  |  |  |  |  |  | *Appropriate with conditions*- PCA with single factor structure, internal consistency >.70, r=-.20 with SensOR. |
| Tavassoli et al., 2014 | 24-51 | 42 | 196=ASD;163=TD | + |  |  | + | + | ? |  |
| Sensory Processing & Self-Regulation Checklist (SPSRC-English) |  |  |  |  |  |  |  |  |  | *Unsupported/*  *Insufficient*-small ASD sample, ICC<.70 for some subscales, correlational coefficient <.70. |
| Gomez et al., 2021 | 4-12 | 52 | 30=DD (6 =ASD);  164=TD | ? | ? |  |  | ? | ? |  |
| Sensory Reactivity in Autism Spectrum (SR-AS) |  |  |  |  |  |  |  |  |  | *Appropriate with Conditions*- sufficient reliability coefficient and factor structure fit, content validity evaluated for experts and target population. Appropriate for use with high-functioning autistic adults. |
| Elwin et al, 2016 | 18-65 | 41 | 71=ASD; 162=TD | + |  |  | + | + |  |  |
| Sensory Assessment for Neurodevelopmental Disorders (SAND) |  |  |  |  |  |  |  |  |  | *Appropriate with Conditions*-Item consistency for subscales not evaluated, content validity evaluated with experts but no target population. |
| Siper et al., 2017 | 2-12 | 56 | 44=ASD; 36=TD | +/- | + | + | ? |  | + |  |
| Sensory Behavior Questionnaire (SBQ) |  |  |  |  |  |  |  |  |  | *Unsupported/*  *Insufficient* – Correlation coefficients >.70, content validity stated by expert opinion without evaluation. |
| Neil et al., 2017 | 6-17 | 68 | 66=ASD; 70=TD | + |  |  | ? |  | + |  |
| Sensory Processing Scales Inventory (SP-Scales Inventory) |  |  |  |  |  |  |  |  |  | Unsupported/*Insufficient*- unclear if SMD sample includes ASD, forced 3-factor loading accounted for 24.3% of variance, content validity stated by expert opinion without evaluation. |
| Schoen et al., 2017 | 4-18 | 61 | 267=SMD  140-=TD | ? |  |  | ? | ? |  |  |
|  |  |  |  |  |  |  |  |  |  |  |
| Brain Body Center Sensory Scales (BBCSS) |  |  |  |  |  |  |  |  |  | *Appropriate with Conditions*-Some subscale consistency <.70, retest reliability <.70, no reported expert evaluation of content validity. |
| Kolacz et al., 2018 | 5-58 | 90 | 333=FXS (136 ASD features) | +/- | + | +/- | ? | + | +/- |  |
| Sensory Sensitivity Scales (SeSS) |  |  |  |  |  |  |  |  |  | *Unsupported/*  *Insufficient*-No ASD sample for CFA and reliability, no evaluation of comprehensibility. |
| Aykan et al., 2020 | 18-28 | Sample 1=55  Sample 2=44 | Sample 1, 896=TD;  Sample 2, 930=TD | ? |  |  | ? | ? | ? |  |

Note. +, sufficient evidence; +/-, sufficient for one or more, but not all subscales; –, contrary evidence; ?, unknown, owing to poor methodological quality, or mixed results; blank cell owing to no evidence available. TD=typically developing, ASD=autism spectrum disorder, NR= not reported, DD=developmental delay, MR=Mental retardation, PDD=pervasive developmental disability, ICC=Intra-class coefficient, OR= over-responsive, SMD=Sensory Modulation Disorder, CFA=confirmatory factor analysis, FXS=fragile X syndrome, PCA=principal component analysis.
